# Scalable design of orthogonal DNA barcode libraries

**DOI:** 10.1038/s43588-024-00646-z

**Published:** 2024-06-07

**Authors:** Gokul Gowri, Kuanwei Sheng, Peng Yin

**Affiliations:** 1 Department of Systems Biology, Harvard Medical School, Boston, MA USA; 2 Wyss Institute for Biologically Inspired Engineering at Harvard University, Boston, MA USA

**Keywords:** Software, Applied mathematics, Biomedical engineering

## Abstract

Orthogonal DNA barcode library design is an essential task in bioengineering. Here we present seqwalk, an efficient method for designing barcode libraries that satisfy a sequence symmetry minimization (SSM) heuristic for orthogonality, with theoretical guarantees of maximal or near-maximal library size under certain design constraints. Seqwalk encodes SSM constraints in a de Bruijn graph representation of sequence space, enabling the application of recent advances in discrete mathematics^1^ to the problem of orthogonal sequence design. We demonstrate the scalability of seqwalk by designing a library of >10^6^ SSM-satisfying barcode sequences in less than 20 s on a standard laptop.

## Abstract (web summary)

Seqwalk is a scalable method for designing orthogonal DNA barcode libraries, producing one million barcodes in 20 s on a standard laptop.

## Main

Orthogonal DNA barcode libraries are widely used in modern biotechnology. For example, orthogonal sequences are used to barcode protein targets in DNA-based bioimaging^2^, to label RNA molecules in individual cells for single-cell studies^3^ and to program the assembly of components in a synthesis process^4^, among many other applications^5–9^. The number of addressable features (cells, protein targets and so on) in these methods is dependent on the size of the orthogonal DNA sequence library that is used. As such, the problem of designing large orthogonal DNA sequence libraries appears across many areas of study in bioengineering.

Depending on the specific application, there are different approaches to designing orthogonal sequence libraries. One powerful approach is using physical models to design sequences such that off-target interactions are thermodynamically unfavorable^10^. Currently, scaling thermodynamic design tools to massive libraries (for example, exceeding 10^5^ nucleotides) or exhaustive searches of large sequence space (for example, all 4^25^ possible 25-nt sequences) is prohibitively computationally expensive^11^.

One widely used alternative is sequence symmetry minimization (SSM)^4,7,12–17^. A set of sequences is considered to satisfy SSM for length *k* if no subsequence of length *k* appears more than one time in the set. In technologies with sequencing-based barcode readouts, satisfying SSM decreases the likelihood of incorrectly assigning barcodes^17^. In technologies with hybridization-based barcode readouts, satisfying SSM decreases the probability of off-target binding^14,15^. It is important to note that, while an informative heuristic, SSM does not explicitly capture thermodynamic properties of sequences, and cannot guarantee low off-target binding energies (Supplementary Notes 6 and 7).

Sequence symmetry has the appealing property that it can be mathematically represented using de Bruijn graphs^18–20^. In this Brief Communication, we show that this graph representation of sequence space enables a massively scalable approach to DNA barcode design. We build on recent advances in discrete mathematics^1,21^ to develop seqwalk, an efficient tool for designing SSM-satisfying barcode libraries, and provide theoretical bounds on orthogonal library size under various design constraints. We provide accessible software implementations of seqwalk and show that it is capable of designing >10^6^ 25-nt barcode sequences in less than 20 s on a single standard central processing unit (CPU) core, with provable guarantees of maximal library size under an SSM constraint.

## *k*-mer graphs for orthogonal sequence design

The key observation underlying seqwalk is that orthogonality constraints in sequence design problems can be naturally encoded in de Bruijn graph representations of sequence space^18–20^. De Bruijn graphs, also known as *k*-mer graphs, are sequence representations that have been well studied in discrete mathematics^1,21,22^. A *k*-mer is a length *k* sequence. A *k*-mer graph has all possible *k*-mers as nodes, and edges between *k*-mers that overlap by *k* − 1 symbols. In particular, if a *k*-mer *k*_1_ can be transformed into a *k*-mer *k*_2_ by removing its first symbol and appending a symbol, then there is a directed edge from *k*_1_ to *k*_2_.

On a *k*-mer graph, a length *L* sequence can be represented as a path over *L* − *k* + 1 nodes. The traversed nodes will correspond to each *k*-mer that appears in the sequence. A set of sequences that can be represented as nonintersecting paths on a *k*-mer graph share no common *k*-mers and, thus, satisfy SSM for the corresponding *k*. This points toward a method for generating sequences that implicitly satisfy SSM for length *k*: one can simply select several nonintersecting paths on a *k*-mer graph. One way to produce nonintersecting paths on a graph is to take a single self-avoiding walk and then partition this walk into multiple nonintersecting paths. The longest possible self-avoiding walks on a graph are Hamiltonian paths, which visit every node of the graph exactly one time. A partitioned Hamiltonian path will result in sequences that fully occupy *k*-mer space and, thus, yield maximally sized orthogonal sequence libraries.

**Fig. 1:**
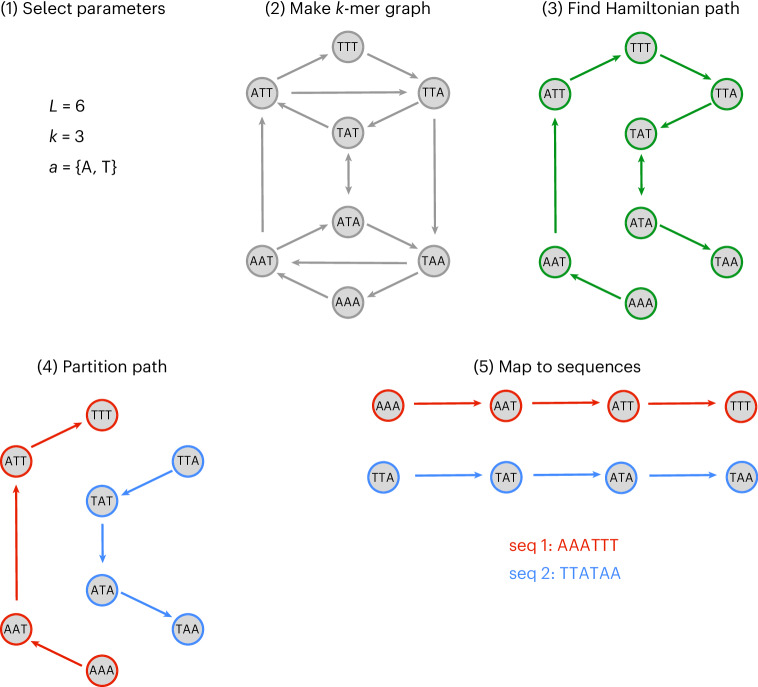
Workflow of graph-based sequence design algorithm. (1) Select length of desired sequences *L*, length of prevented substrings (SSM constraint) *k*, and allowed alphabet *a*. (2) Represent sequence space using a *k*-mer graph. (3) Select a Hamiltonian path in *k*-mer graph. (4) Partition the selected Hamiltonian path into fragments with *L* − *k* + 1 nodes. (5) Map fragments to corresponding sequences.

In seqwalk, we apply a recently discovered mathematical technique for traversing de Bruijn graphs, which yields Hamiltonian paths in amortized *O*(1) time and memory per node^1^, to efficiently and scalably design orthogonal sequence libraries (Fig. 1). While finding Hamiltonian paths in arbitrary graphs is computationally hard^23^, the mathematical structure of de Bruijn graphs enables efficient identification of Hamiltonian paths. The core algorithm is simple: our implementation requires less than 100 lines of code, including output formatting.

## Performance benchmarks

To understand the practical relevance of the efficiency of seqwalk, we compare its time and memory efficiency with DeLOB^7^, an existing approach for large orthogonal library design. In brief, DeLOB begins with a large candidate library of random sequences, uses BLAST^24^ to identify pairs of sequences that violate SSM with *k* = 12, then chooses a subset of sequences that do not violate SSM. While DeLOB candidate libraries can be refined on the basis of sequence design constraints (such as melting temperature or lack of intramolecular secondary structure), for the purpose of benchmarking, we reimplemented DeLOB with unconstrained candidate libraries of random sequences (Methods).

**Fig. 2:**
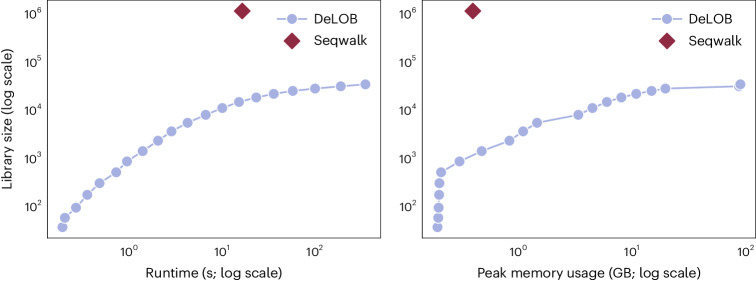
Time and memory efficiency of seqwalk in comparison with a reimplementation of the DeLOB method, described in ref. ^7^, for the problem of designing 25-nt barcodes satisfying SSM *k* = 12, with no additional sequence constraints. DeLOB was run with varying choices for number of candidate sequences from 10^3^ to 10^5^, resulting in a range of computational costs and library sizes. All computations were performed using a single CPU core. Source data.

We run DeLOB with various numbers of candidate sequences and compare this with seqwalk run with an SSM constraint of *k* = 12. We find that seqwalk produces about two orders of magnitude more sequences than DeLOB run for a similar time (Fig. 2). In our benchmarking setup, DeLOB has a peak memory usage of nearly 100 GB to design about 3.6 × 10^4^ sequences, making it incompatible with current personal computing hardware. In comparison, seqwalk has peak memory usage of less than 1 GB and produces over 10^6^ sequences. In summary, seqwalk is capable of efficiently producing SSM-satisfying sequence libraries, requiring only standard personal computing hardware to design libraries exceeding 10^6^ sequences in less than 30 s.

Seqwalk’s exhaustive traversal of sequence space is also useful for designing small orthogonal libraries with minimal sequence symmetry. We demonstrate this by comparing a seqwalk library with a widely used multiplexing barcode library for a single-cell RNA sequencing method, MULTI-seq^25^. The original MULTI-seq library consists of nine distinct 8-nt barcodes, designed to have pairwise Hamming distances ≥3. This design strategy yields barcodes with high sequence symmetry, resulting in barcode ambiguity that may give rise to experimental artifacts^17^. The seqwalk equivalent of this library, designed with the smallest *k* to yield at least nine sequences (*k* = 3), has minimal barcode ambiguity, lower homopolymer prevalence, improved pairwise Hamming distances and similar guanine-cytosine (GC) diversity (Supplementary Note 5 and Supplementary Table 1).

## Sequence design under additional constraints

In many applications, there are additional constraints to orthogonal sequence libraries beyond crosstalk between barcodes. One common constraint is the prevention of crosstalk with reverse complements of sequences in the library. For sequence design under this constraint, seqwalk integrates two approaches: a filtering approach, and an adaptation of the Hierholzer algorithm^26^ for four-letter libraries with odd *k* (Methods).

Seqwalk design can also consider other common constraints such as requiring GC content or melting temperature within a window, the absence of specific sequence patterns and the absence of substantial secondary structure. We provide efficient algorithms for filtering seqwalk libraries for these characteristics (Supplementary Notes 1–4). We find that three-letter seqwalk libraries are particularly amenable to such filtering, as they have sequences with lower variance in GC content and melting temperature (Supplementary Figs. 1 and 2), low prevalence of secondary structure (Supplementary Fig. 3) and less crosstalk with reverse complements (Methods).

## Theoretical results

SSM-satisfying sequence libraries designed by the partitioning of a Hamiltonian path (such as in seqwalk) are maximally sized. This can be trivially proven by contradiction, by noting that every possible *k*-mer in the sequence space appears in the library. If there existed a larger library of SSM-satisfying sequences, it would use a larger number of *k*-mers and, thus, would repeat *k*-mers and not satisfy SSM (Methods).

Fundamental results about de Bruijn graphs^22^ almost directly yield a closed form expression for the number of sequences in seqwalk libraries under different design parameters. For alphabet size *m*, sequence length *L* and SSM constraint *k*, the number of possible orthogonal sequences *N* is the number of nodes in the *k*-mer graph divided by the number of nodes required to represent a sequence of length *L*. More precisely,

1 $$N=\left\lfloor \frac{{m}^{\rm{k}}}{L-k+1}\right\rfloor.$$

**Extended Data Fig. 1:**
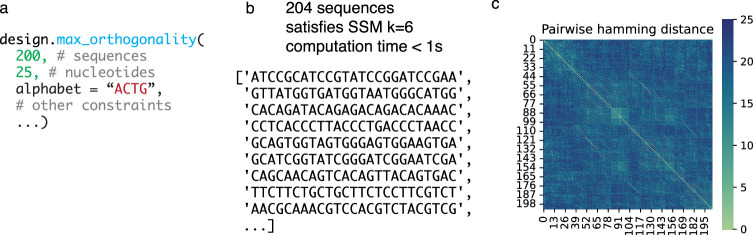
Depiction of seqwalk software package. (a) Example design code which produces a library of at least 200 25nt sequences with maximal orthogonality according to the SSM heuristic. (b) Output of example design code. (c) Crosstalk analysis of designed library using Hamming distance. Each row/column represents a sequence, and each entry is colored by Hamming distance. Source data.

This theoretical result has practical relevance. For a practitioner who wishes to design a certain number of sequences, the strongest possible SSM constraint (that is, the smallest possible *k*) can be determined using the relationship between *k* and *N*. Given a desired library size of *N*_*d*_, sequence length *L* and alphabet size *m*, we can choose the smallest *k* such that *N* ≥ *N*_*d*_. Designing a library using the resulting *k* value yields a maximally orthogonal (as defined by SSM) library with the desired number of sequences. This function is implemented in the seqwalk software library and named max_orthogonality (Extended Data Fig. 1).

While the proof of maximal library size does not hold under additional design constraints (such as reverse complement prevention, GC content filtering and so on), we can estimate or exactly state useful lower bounds on the size of seqwalk libraries after downstream filtering. For example, we can place lower bounds on the number of sequences present after a filtering for a specific sequence pattern of length *p* ≤ *k*. The number of *k*-mers containing a specific pattern of length *p* is

2 $${K}_{\rm{p}}\le (k-p+1)\times {m}^{\rm{k-p}},$$

where *m* is the size of the alphabet. Since no *k*-mer appears in more than one sequence in the library, we must remove at most *K*_p_ sequences from our library to remove all sequences containing a pattern of length *p*. As such, the size of the filtered library, *N*_p_, is

3 $${N}_{\rm{p}}\ge N-{K}_{\rm{p}}.$$

Such lower bounds are simple to determine for practically relevant pattern constraints, such as the prevention of homopolymeric regions (Supplementary Note 4). For certain choices of *k*, *L* and *p*, the size of pattern-free seqwalk libraries is near identical to the maximum possible library size under no pattern constraint. For example, for patterns with length *p* = *k*, at most one sequence is removed per pattern.

Additionally, we derive a lower bound on the size of seqwalk libraries upon filtering for orthogonality with reverse complements (Methods). For the case of three-letter libraries with odd *k*, we show that the size of a seqwalk library that satisfies orthogonality with reverse complements, *N*_rc_, can be bounded by

4 $${N}_{{\mathrm{rc}}}\ge N-{2}^{\rm{k-1}}.$$

The size of seqwalk libraries under GC content constraints is not as easily determined analytically. However, empirical results show that seqwalk libraries have consistent distributions of GC content, resembling the binomial distribution expected of uniformly random sequences (Supplementary Note 1). As such, these distributions can be used to estimate the size of seqwalk libraries under GC content constraints.

## Implementation as a software tool

We have implemented the seqwalk algorithm and additional filtering tools in a ‘pip’ distributed Python package (seqwalk, documented at seqwalk.readthedocs.io). Additionally, we have developed an interactive, code-free, web-based seqwalk interface in a publicly accessible Google Colaboratory notebook (link on seqwalk.readthedocs.io), based on a Julia implementation. While the Julia implementation is faster, the Python implementation and package allow for easier incorporation with the existing ecosystem of tools for sequence design and analysis. We envision the use of seqwalk as a part of a sequence design pipeline, with downstream filtering (experimental validation, genomic homology filtering and so on) as necessary for specific application contexts. Due to the simplicity of the underlying algorithms, we expect that others can implement our design method in other settings and modify it as necessary for different design pipelines.

## Discussion

In this paper, we introduced seqwalk, a method for scalably designing DNA barcode libraries that satisfy SSM constraints. Seqwalk enables the design of SSM-satisfying libraries consisting of millions of sequences, using only standard personal computing hardware.

While seqwalk can be applied to many design problems, its use of the SSM heuristic makes it more directly applicable in certain experimental contexts. In particular, seqwalk is well suited for problems where nuanced biophysical properties (that is, exact Δ*G*) do not need to be tightly controlled (Supplementary Notes 6 and 7). In settings where biophysical or other experimental design constraints are strong, seqwalk can be used upstream of other design tools as a way to quickly constrain design space on the basis of an SSM heuristic. We expect that seqwalk can be valuable, either alone or in conjunction with other sequence design tools, for the rapidly growing class of high-throughput biological methods that use synthetic DNA sequences as barcodes for different biomolecular features (that is, samples, cells, protein targets, plasmids and so on).

Additionally, the theoretical guarantees on the size of seqwalk libraries can be used to guide design choices in experimental method development. Using the results presented in this paper, one can quickly assess tradeoffs between design parameters and orthogonal sequence library size.

At least two threads of future investigation are raised by this work. First, the graph representation used in seqwalk only captures orthogonality as defined by SSM. Is it possible to generalize the approach to other notions of orthogonality, such as those defined by physical models? Second, as SSM remains an appealing orthogonality heuristic for its tractability, can we precisely identify experimental settings where it is insufficient?

Graph representations of sequences are commonly used to describe naturally occurring biological sequences^27^. There is growing interest in sequence representations amenable to design tasks, in addition to descriptive tasks^28,29^. With seqwalk, we demonstrate that graph-based sequence representations enable massive efficiency improvements in SSM-satisfying sequence library design.

## Methods

### Clarifying notions of orthogonality

Here, we will try to be more precise about what we mean by ‘orthogonality’ and ‘crosstalk’. We will separate the discussion for two broad application categories of DNA barcodes: sequencing-based and hybridization-based.

For sequencing-based barcodes, we consider two barcodes (A and B) to have crosstalk if they cannot be easily disambiguated on the basis of sequencing readout of the barcode. In other words, if barcode A and barcode B can be distorted into the same sequence through the process of library preparation, sequencing and alignment.

For hybridization-based barcodes, we consider two sequences (A and B) to have crosstalk if they can stably hybridize with each other’s reverse complements. In other words, if a complex between A and B* or A* and B is likely to form with experimentally relevant propensity, we consider A and B to have crosstalk.

If we think of A and B as probes, with A* and B* being their respective targets, we consider crosstalk to be the binding of a probe to an incorrect target. We do not by default consider binding between A and B to be crosstalk.

For many, but not all, applications, this is a sufficient characterization of crosstalk. In the case of multiplexed DNA exchange imaging, a single probe (referred to as imager in the multiplexed imaging literature), rather than a pool of probes, can be present in a sample at a given time^2^. As such, one need not consider binding between probes. Analogously, in DNA similarity search, a single ‘query’ probe is used to bind ‘target’ strands, so preventing binding between probe strands is not necessary^30^.

In some applications, where orthogonal sequence libraries and their reverse complements are mixed together in a single reaction, such as in multiplexed PCR^31^, a stronger definition of crosstalk is required. We call this orthogonality including reverse complements, where A and B have crosstalk if any pair of A, A*, B, B* have substantial binding (other than the desired A with A*, and B with B*).

For all cases above, SSM is an applicable heuristic for orthogonality. While other heuristics are stronger for certain applications (Supplementary Notes 6 and 7), in this paper, we consider only SSM, as it enables scalable sequence design via mathematical abstraction.

### Proof of maximal library size under SSM constraints

#### Definitions

- Sequence library: set of sequences of length *L* over alphabet of size *m*
- *k*-mer: subsequence of length *k*
- SSM satisfied for length *k*: no subsequence of length *k* appears more than once, for *k* ≤ *L*
- Maximally sized SSM sequence library: a sequence library satisfying SSM for length *k* with size such that no larger sequence library satisfying SSM for length *k* exists.

#### Lemma 1

A maximally sized sequence library that satisfies SSM for length *k* contains at most *m*^k^ distinct *k*-mers.

#### Proof of Lemma 1

Assume for the sake of contradiction that there exists an SSM satisfying library for length *k*, which has *K* > *m*^k^ *k*-mers. Since there are only *m*^k^ possible *k*-mers, by the pigeonhole principle, at least one *k*-mer must appear >1 time in the library. Since a *k*-mer appears more than once in the library, it does not satisfy SSM. We have arrived at a contradiction.

#### Theorem 1

A sequence library generated by the partitioning of a Hamiltonian path in a *k* de Bruijn graph is a maximally sized SSM sequence library for length *k*.

#### Proof of Theorem 1

By definition, the number of *k*-mers in such a library is equal to the number of nodes in the corresponding de Bruijn graph. The number of nodes in the de Bruijn graph, by definition, is *m*^k^. By Lemma 1, a maximally sized sequence library that satisfies SSM for length contains at most *m*^k^ k-mers. Thus, no larger SSM satisfying library exists.

### Orthogonality with reverse complements

In the seqwalk package, we implement three different strategies for orthogonal sequence design that considers reverse complementarity.

For the case of three-letter alphabets and odd SSM *k* values, we describe an efficient algorithm for filtering out reverse complementary sequences. Without loss of generality, consider the case of sequences constructed with an A, C, T library.

We want a library with no repeated *k*-mers, and no *k*-mers whose reverse complement also appears in the library. *k*-mers containing C cannot have their reverse complements also appear in the library, since the library will not contain G. So, we only need to consider *k*-mers composed entirely of A and T.

To use *k*-mers whose reverse complement will not appear in the library, we partition all AT *k*-mers into two sets, such that the reverse complement of each sequence in one set appears in the other set. Then, we can remove all sequences containing *k*-mers from one partition. Thus, the reverse complements of any *k*-mers that appear in the library will not be present.

For odd *k*, we can easily find a partitioning by noting that the middle base in the *k*-mer must be different from its reverse complement. For example, in a 5-mer, the third base can never be the same as the third base of its reverse complement. So, we can simply divide *k*-mers into two sets according to the identity of its middle base.

Using this approach, we can easily lower bound the size of a resulting library will be upon filtering. We know that there are 2^k^ *k*-mers consisting entirely of A and T. Half of these *k*-mers will have A as the middle base. At most, we will remove one sequence from the library for each *k*-mer with A as the middle base. As such, we can lower-bound the number of sequences upon reverse complementarity filtering, *N*_rc_ using

$${N}_{{\mathrm{rc}}}\ge N-{2}^{\rm{k-1}}.$$

This theoretical result indicates that SeqWalk still produces relatively large sequence libraries upon such filtering. For example, for the case of 25-nt barcodes with three-letter code, SSM *k* = 13, and removal of reverse complements, we will have a sequence library with at least ${N}_{{\mathrm{rc}}}\ge \frac{{3}^{13}}{13}-{2}^{12}=1.18544\times 1{0}^{5}$ sequences.

In the case of a four-letter alphabet, filtering is more difficult because we cannot constrain reverse complementary *k*-mers to AT sequences. For odd-*k* and four-letter codes, we use a modification of the Hierholzer algorithm, in which we mark both the visited *k*-mer and its reverse complement ‘visited’ during traversal. This method requires keeping track of visited nodes and, as such, is less time/memory efficient than the shift rule traversal. Our implementation can be found in the adapted_hierholzer function in the generation module of the seqwalk source code.

For even-*k*, we implement a simple hashing-based approach to filter out reverse complements. We iterate through each sequence in a SSM-satisfying (without considering reverse complements) library, and if it has a *k*-mer that matches the reverse complements of previous sequences in the library, we remove the sequence from the library.

### Benchmarking DeLOB performance

DeLOB^7^ and seqwalk design libraries using similar, but not identical, design constraints. DeLOB uses the presence BLAST high-score segment pairings (HSP) of length 13 or more as heuristic for crosstalk. Based on the BLAST parameters used in DeLOB, an HSP must contain at least 11 bases of exact match. This means the SSM of *k* = 12 is at least as strong of an orthogonality criterion as used in DeLOB. As such, we compare DeLOB to seqwalk libraries designed with the *k* = 12 constraint. DeLOB, as presented in ref. ^7^, also filters sequences for melting temperature, secondary structure and absence of restriction sites. To more directly compare DeLOB and seqwalk, we reimplemented DeLOB with no additional sequence filtering beyond orthogonality and used seqwalk with no additional sequence filtering.

### Reporting summary

Further information on research design is available in the Nature Portfolio Reporting Summary linked to this article.

### Supplementary information

Supplementary Information Supplementary Figs. 1–7, Table 1 and Notes 1–7.

Reporting Summary

### Source data

Source Data Fig. 2 Numerical performance data for sequence design software.

Source Data Extended Data Fig. 1 Hamming distance matrix of depicted sequence library.

## Data Availability

Source data for Fig. 2 and Extended Data Fig. 1 are made available with this manuscript. The numerical data supporting the findings of this paper are provided in the source data files, and the sequences themselves can be generated by running our software. Source data are provided with this paper.

## Code availability

The code to reproduce all analysis from this paper can be found via GitHub at github.com/ggdna/seqwalk_paper_reproducibility. The software library can be found via Zenodo at 10.5281/zenodo.10932482 ref. ^32^ and installed from https://pypi.org/project/seqwalk/.
