# Supplementary material for: Scalable design of orthogonal DNA barcode libraries
Source: Nat Comput Sci. 2024 Jun 7;4(6):423–8. doi: 10.1038/s43588-024-00646-z (PMC11208133; doi:10.1038/s43588-024-00646-z)
Supplement: Supplementary file 1 — Supplementary Figs. 1–7, Table 1 and Notes 1–7. [file 43588_2024_646_MOESM1_ESM.pdf]

nature computational science

Supplementary information

https://doi.org/10.1038/s43588-024-00646-z

Scalable design of orthogonal DNA barcode libraries

In the format provided by the authors and unedited

Supplementary information: Scalable design of orthogonal DNA barcode libraries

Gokul Gowri, Kuanwei Sheng, Peng Yin

Supplementary note 1: GC content distributions in SeqWalk libraries

While we do not have a rigorous proof of this, we find empirically that the distributions of GC content in SeqWalk libraries is similar to that of totally random sequences. We expect, in a 4 letter alphabet, to have GC content that is binomially distributed with p=0.5 and n=L (where L is the sequence length). In a 3 letter alphabet (ACT or AGT) we expect to have GC content binomially distributed with $p = \frac{1}{3}$ and $n = L$.

The variance of a binomial is $\sigma^2(n,p) = p * (1-p) * n$, with $p \in [0,1]$. Since $p * (1-p)$ is maximized for $p = 0.5$, the variance of GC content is highest for the case of a 4 letter alphabet. This is in line with empirical results, shown in Supplementary Figure 1.

Since the GC content of 3 letter alphabet libraries is lower, a tighter window of GC content constraints can be used to obtain the same number of sequences (assuming that the extreme GC content sequences are those to be filtered out).

For filtering libraries for GC content, a straightforward algorithm, such as the one below, is efficient (O(n) in the size of the library).

```
function gc_filter(SeqWalk_library, gc_min, gc_max)

    filtered_library = []

    for seq in SeqWalk_library
        gc_content = gc(seq)
        if gc_min < gc_content < gc_max
            push!(filtered_library, seq)
        end
    end

end
```

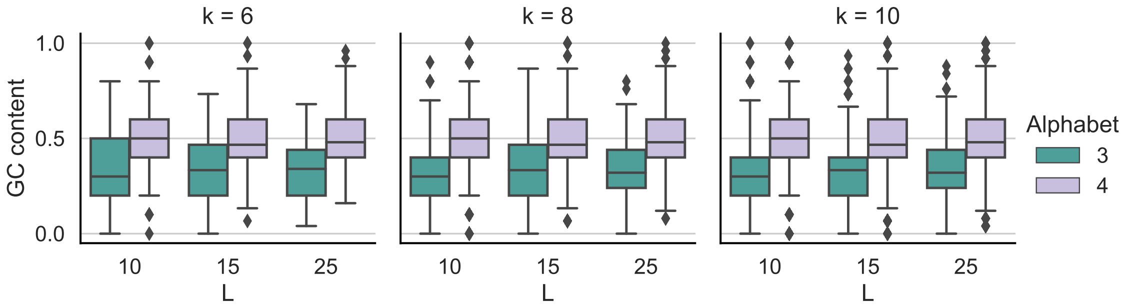

Supplementary Figure 1. GC content distributions in various SeqWalk libraries. The 3 letter libraries are designed using {A, C, T}, while the 4 letter libraries use {A, C, T, G}. Boxes indicate quartiles, and whiskers extend to data within 1.5 times the interquartile range (IQR) of the lower and upper quartiles. Sample size information: n=145 3-letter sequences and n=819 4-letter sequences were analysed for k=6, L=10; n=72 3-letter sequences and n=409 4-letter sequences were analysed for k=6, L=15; n=36 3-letter sequences and n=204 4-letter sequences were analysed for k=6, L=25; n=2187 3-letter sequences and n=21845 4-letter sequences were analysed for k=8, L=10; n=820 3-letter sequences and n=8192 4-letter sequences were analysed for k=8, L=15; n=364 3-letter sequences and n=3640 4-letter sequences were analysed for k=8, L=25; n=9841 3-letter sequences and n=174762 4-letter sequences were analysed for k=10, L=15; n=3690 3-letter sequences and n=65536 4-letter sequences were analysed for k=10, L=25.

Supplementary note 2: Melting temperature distribution in SeqWalk libraries

Empirically, we find that the distribution of melting temps in SeqWalk libraries is qualitatively similar to that of GC content (Supplementary Figure 1). Melting temperature is calculated using default conditions of the BioPython SeqUtils package. As expected, the mean melting temperatures of SeqWalk libraries with 'ACT' alphabet is lower than that of libraries with 'ACGT' alphabet.

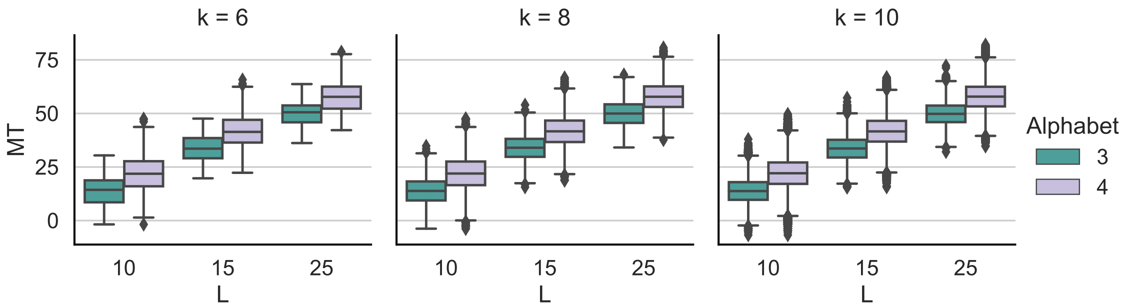

Supplementary Figure 2. Melting temperature distributions in various SeqWalk libraries. The 3 letter libraries are designed using {A, C, T}, while the 4 letter libraries use {A, C, T, G}. Boxes indicate quartiles, and whiskers extend to data within 1.5 times the interquartile range (IQR) of the lower and upper quartiles. Sample size information: n=145 3-letter sequences and n=819 4-letter sequences were analysed for k=6, L=10; n=72 3-letter sequences and n=409 4-letter sequences were analysed for k=6, L=15; n=36 3-letter sequences and n=204 4-letter sequences were analysed for k=6, L=25; n=2187 3-letter sequences and n=21845 4-letter sequences were analysed for k=8, L=10; n=820 3-letter sequences and n=8192 4-letter sequences were analysed for k=8, L=15; n=364 3-letter sequences and n=3640 4-letter sequences were analysed for k=8, L=25; n=9841 3-letter sequences and n=174762 4-letter sequences were analysed for k=10, L=15; n=3690 3-letter sequences and n=65536 4-letter sequences were analysed for k=10, L=25.

Supplementary note 3: Secondary structure in SeqWalk libraries

Empirically, we find that secondary structure is very uncommon in SeqWalk libraries constructed with {A, C, T} alphabet. We use percentage of paired bases in the MFE structure as a measure of secondary structure prevalence in a sequence. We are aware that this is not an ideal measure [1], but we use it as it is convenient to compute and useful for relative comparisons.

To filter sequences for secondary structure, we can again use a straightforward algorithm, such as the one below, which runs in O(n) time in the size of the library.

```
function SS_filter(SeqWalk_library, SS_threshold)

    filtered_library = []

    for seq in SeqWalk_library
        ss = NUPACK_MFE_Unpaired_fraction(seq)
        if ss < SS_threshold
            push!(filtered_library, seq)
        end
    end

end
```

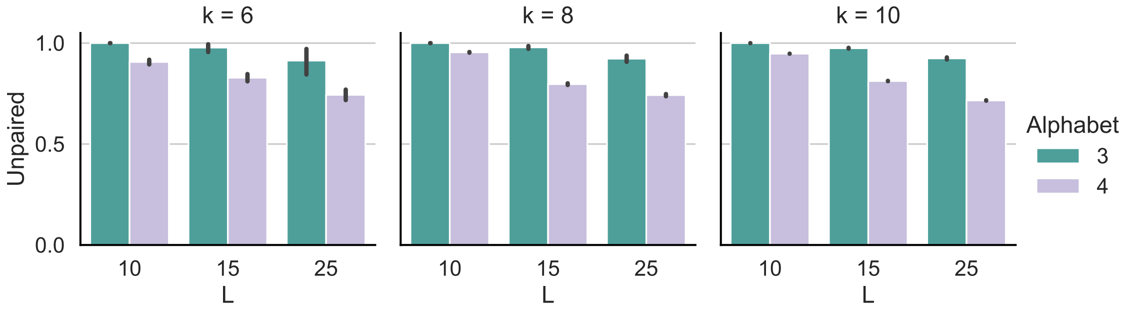

Supplementary Figure 3. Distribution of fraction of unpaired bases in MFE structures in various SeqWalk libraries at 22C, 5mM Na+, 2mM Mg2+. An "Unpaired" value of 1 indicates no bound bases in the MFE structure of a sequence. The 3 letter libraries are designed using {A, C, T}, while the 4 letter libraries use {A, C, T, G}. Error bars indicate 95% confidence interval computed from n=1000 bootstrap samples. Sample size information: n=145 3-letter sequences and n=819 4-letter sequences were analysed for k=6, L=10; n=72 3-letter sequences and n=409 4-letter sequences were analysed for k=6, L=15; n=36 3-letter sequences and n=204 4-letter sequences were analysed for k=6, L=25; n=2187 3-letter sequences and n=21845 4-letter sequences were analysed for k=8, L=10; n=820 3-letter sequences and n=8192 4-letter sequences were analysed for k=8, L=15; n=364 3-letter sequences and n=3640 4-letter sequences were analysed for k=8, L=25; n=9841 3-letter sequences and n=174762 4-letter sequences were analysed for k=10, L=15; n=3690 3-letter sequences and n=65536 4-letter sequences were analysed for k=10, L=25.

Supplementary note 4: Preventing specific sequence patterns

We can place lower bounds on the number of sequences present after a filtering for a specific sequence pattern of length $p \leq k$. The number of k-mers containing a specific pattern of length p is

$$K_p \leq (k - p + 1) * m^{k-p}$$

where m is the size of the alphabet. Since no k-mer appears in more than one sequence in the library, we must remove at most K_p sequences from our library to remove all sequences containing a pattern of length p. As such, the size of the filtered library, N_p, is

$$N_p \geq N - K_p$$

Such lower bounds are simple to determine for practically relevant pattern constraints, such as the prevention of homopolymeric regions.

For example, we can consider the case of preventing 4G regions, as well as 4N (any of 4A, 4T, 4C, 4G) regions. To lower bound the number of sequences after removing all 4N regions, we can use

$$N_{4N} \geq N - (K_{4A} + K_{4T} + K_{4C} + K_{4G})$$

Below, we see plots of these bounds for various k and L.

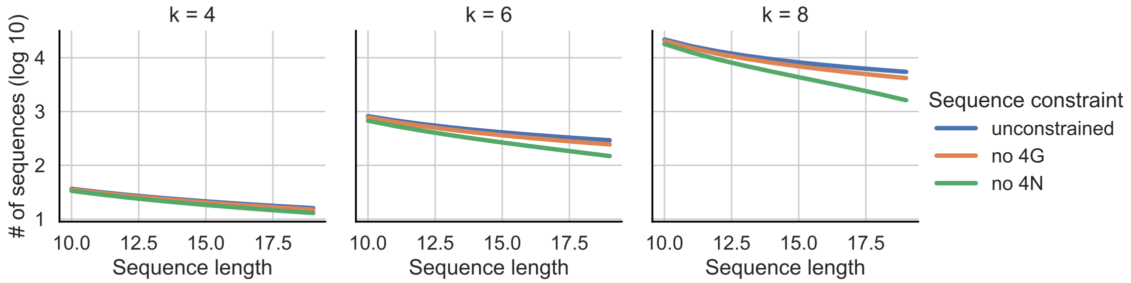

Supplementary Figure 4. Lower bounds on library size for various design problems under different sequence pattern prevention constraints. In particular, we plot lower bounds for 4 letter SeqWalk libraries preventing 4G and preventing all 4N, in comparison to libraries with no pattern prevention constraints.

Supplementary note 5: Comparing MULTI-seq barcodes and a SeqWalk library

Qcbc is an open-source software tool for validating orthogonal sequencing barcode libraries [2]. Qcbc has been used to reveal experimental artifacts in the Multiseq multiplexed scRNA-seq assay, due to barcode ambiguity [2]. We generate a seqwalk library of the same size and length as the Multiseq library, and use Qcbc to compare the quality of the libraries. Qcbc considers four quality metrics of barcode libraries: (1) barcode ambiguity for values of l, which is the number of barcodes which share a subsequence of at least length l with at least one other barcode, (2) pairwise Hamming distances, (3) nucleotide diversity and (4) homopolymer frequency. For (1) and (4), low values are better, and for (2) and (3), high values are better. The SeqWalk library has a 100% reduction in barcode ambiguity for $l > 2$ (as a result of satisfying SSM for $k = 3$), and improves pairwise hamming distances and homopolymer frequency, while maintaining comparable nucleotide diversity.

QC metric	SeqWalk	Multiseq
Average homopolymer score	3.3	4.1
Ambiguous barcodes, l=3	0	9
Ambiguous barcodes, l=4	0	5
Ambiguous barcodes, l=5	0	2
Ambiguous barcodes, l=7	0	2
Pairwise distance	5.8	5.7
Nucleotide entropy	0.55	0.56

Supplementary Table 1. QCBC outputs for Seqwalk and Multiseq barcode libraries.

Supplementary note 6: Relationship between SSM and binding energy

While sequence symmetry is a widely used heuristic for orthogonality, many other orthogonality heuristics exist. Several sequence design methods are based on equilibrium thermodynamic analysis of DNA strands. While thermodynamic heuristics and sequence symmetry do not match exactly, we find that they often loosely correlate. Through empirical sequence analysis using NUPACK, we demonstrate that the ΔG of pairs of random 25mer DNA sequences is related to their sequence symmetry (longest common shared subsequence). However, it is clear also that low sequence symmetry does not guarantee weak ΔG.

We simulate $5 * 10^4$ pairs of random 25mers at 22C, 300mM sodium, and estimate their ΔG using NUPACK. We categorize the sequences based on sequence symmetry (k value) and plot empirical cumulative distribution functions (eCDFs) for each category, shown in Supplementary Figure 5. Lower k corresponds clearly to weaker average binding (higher ΔG).

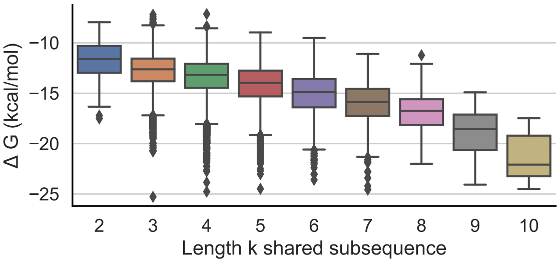

Supplementary Figure 5. eCDFs of NUPACK estimated ΔG of pairs of random 25mer sequences, categorized by sequence symmetry. n=50000 pairs of sequences analysed for each k value. Boxes indicate quartiles, and whiskers extend to data within 1.5 times the interquartile range (IQR) of the lower and upper quartiles.

Supplementary note 7: Comparing the thermodynamic properties of various sequence libraries

SSM, while generally an effective orthogonality heuristic, cannot guarantee weak off-target binding. In this note, we compare the off-target binding energy, as predicted by NUPACK, in a SeqWalk library (designed with $L = 9, k = 6$, and an ACT alphabet) a library of random 9-mer ACT sequences, and the widely used library of 9mers designed in [3], which also uses ACT sequences.

For random pairs of sequences in each library, we compute the off-target binding energy and compare this to the distribution of on-target binding energies in that library. These binding energy predictions are made using NUPACK, for 37C, with 2mM magnesium and 5mM sodium (Supplementary Figure 6). We find that worst case off-target binding is stronger in SeqWalk libraries compared to that of [3], while weaker than random sequences.

Perhaps the more important property for library orthogonality, rather than absolute strength of off-target binding, is that off-target interactions are weaker than on-target interactions. To this end, we estimate the fraction of off-target interactions that are stronger than the weakest on-target interactions (Supplementary Figure 7). We find no such interactions in PER libraries, while we find roughly 4% of off-target interactions stronger than the weakest on-target interactions in a SeqWalk library, and 8% in a random library.

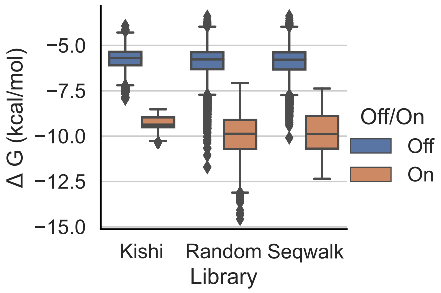

Supplementary Figure 6. Partition function free energy (PFFE) distributions of on-target and off-target interactions in various 9mer libraries. Predictions made using NUPACK, for 37C, with 2mM magnesium and 5mM sodium. Boxes indicate quartiles, and whiskers extend to data within 1.5 times the interquartile range (IQR) of the lower and upper quartiles. n=20000 pairs are sampled for each on-target and off-target distribution in each library.

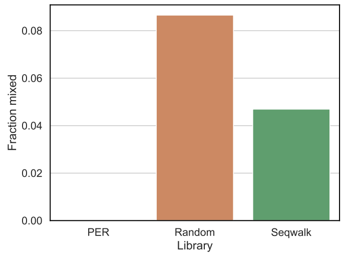

Supplementary Figure 7. Fraction of off-target interactions with stronger predicted binding energy than weakest on-target interaction. We refer to this as “Fraction mixed.”

References

[1] Robert M Dirks, Milo Lin, Erik Winfree, and Niles A Pierce. Paradigms for computational nucleic acid design. *Nucleic Acids Res.*, 32(4):1392–1403, February 2004.

[2] A Sina Booeshaghi, Kyung Hoi Joseph Min, Jase Gehring, and Lior Pachter. Quantifying orthogonal barcodes for sequence census assays. *Bioinform Adv*, 4(1):vbad181, 2024.

[3] Jocelyn Y Kishi, Thomas E Schaus, Nikhil Gopalkrishnan, Feng Xuan, and Peng Yin. Programmable autonomous synthesis of single-stranded DNA. *Nat. Chem.*, 10(2):155–164, February 2018.
